# Quantifying single plasmonic nanostructure far-fields with interferometric and polarimetric k-space microscopy

**DOI:** 10.1038/s41377-018-0059-0

**Published:** 2018-09-12

**Authors:** Ruslan Röhrich, Chris Hoekmeijer, Clara I. Osorio, A. Femius Koenderink

**Affiliations:** 1 Center for Nanophotonics, AMOLF, Science Park 104, 1098 XG Amsterdam, The Netherlands; 2 ARCNL, Science Park 104, 1098 XG Amsterdam, The Netherlands

## Abstract

Optically resonant nanoantennae are key building blocks for metasurfaces, nanosensors, and nanophotonic light sources due to their ability to control the amplitude, phase, directivity, and polarization of scattered light. Here, we report an experimental technique for the full recovery of all degrees of freedom encoded in the far-field radiated by a single nanostructure using a high-NA Fourier microscope equipped with digital off-axis holography. This method enables full decomposition of antenna-physics in its multipole contributions and gives full access to the orbital and spin angular momentum properties of light scattered by single nano-objects. Our results demonstrate these capabilities through a quantitative assessment of the purity of the “selection rules” for orbital angular momentum transfer by plasmonic spiral nanostructures.

## Capturing images of light patterns at the nanoscale

Researchers have developed a three-in-one microscopy technique capable of resolving the various properties of light scattered by a nanosized antenna. The technique has applications in research that studies the behavior of light at the nanoscale (nanophotonics), and in the science and technology of using light to set the standards that define units of measurement (optical metrology). A. Femius Koenderink and colleagues at AMOLF’s Center for Nanophotonics in the Netherlands developed a method, which combines Fourier microscopy, polarimetry, and digital holography to measure the radiation patterns scattered by a single nanosized structure. The technique allows researchers to more clearly observe the physics of, for example, spiral nanostructures, which can generate twisted light. More precise measurements in nanophotonics research could lead to revolutionary applications in telecommunications, computing, and sensing.

## Introduction

A cornerstone of nanophotonics is to precisely control the resonances of individual metallic and dielectric scatterers that form elementary building blocks of nanophotonic devices such as metasurfaces and nanoantennae. The underlying physics is based on the fact that subwavelength geometric tailoring controls the near-field multipolar resonances of nanoscatterers, which in turn allows for a precise manipulation of the amplitude, phase, and polarization of light scattered into the far field. For instance, the past decade has seen the realization of plasmonic nanoantennae^1–3^ to tailor directivity^4–8^ and polarization^9–12^ of scattering and fluorescence, even down to the level of single-photon sources^13^. Additionally, metasurfaces based on metallic and dielectric nanoresonators provide near-arbitrary control over the phase, amplitude, spin, and orbital angular momentum content of transmitted wavefronts^14–17^.

**Fig. 1:**
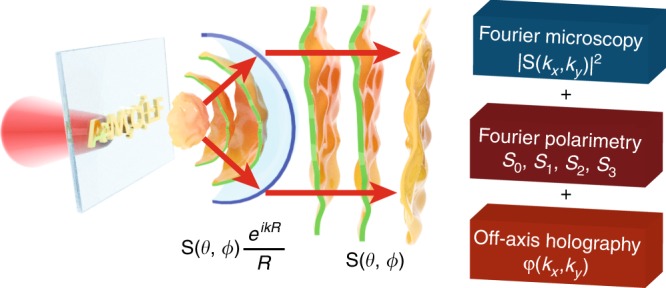
Conceptual sketch. A single nanoantenna radiating a spherical wave with a characteristic phase profile as well as amplitude and polarization. This spherical wave is transformed into a plane wave in the back focal plane of a microscope objective. Measuring the amplitude, polarization, and phase content of the light over all angles enables detailed reconstruction of the antenna physics

Even though a successful design requires a precise understanding of the type of multipolar resonances supported by a nano-object, the complex superposition one can excite, and how these radiate into the far field, such an understanding commonly relies largely on numerical results and is supported only indirectly by experimental evidence. In principle, a measurement of the full polarization, amplitude, and phase of light for each angle in the 4*π* far-field radiation pattern of a nanoantenna enables full decomposition of the antenna’s response in its locally induced multipoles (see Fig. 1). Thus, full field radiation pattern measurement at all angles can enable complete nanoantenna polarizability tomography. In this work, we present a phase- and polarization-resolved Fourier microscope that meets this challenge over the NA spanned by a high-NA microscope objective. We demonstrate the potential of this method by phase resolving the radiation pattern of single spiral-shaped nanoscatterers that generate orbital angular momentum (OAM)^18^. OAM beams have envisioned applications in optical communication technology^19,20^, quantum information processing^21^, and optical manipulation^22^. Since these applications require a precise knowledge of the OAM mode content, the detection and analysis of paraxial OAM beams have been a subject of great interest^23–26^. However, a quantitative assessment of the purity with which plasmonic nanostructures, such as spiral nanostructures, transfer OAM to scattered, spherical waves is still missing. Here, we present such measurement results, which not only demonstrate the capabilities of our technique but are also highly relevant in view of shrinking applications for OAM beams to length scales of, e.g., single-photon emitters.

In addition to opening up new possibilities in the research of nanoantennae and metasurfaces, we envision that the proposed method will have a large impact on optical metrology applications^27^.

## Results

### Experimental set-up

**Fig. 2:**
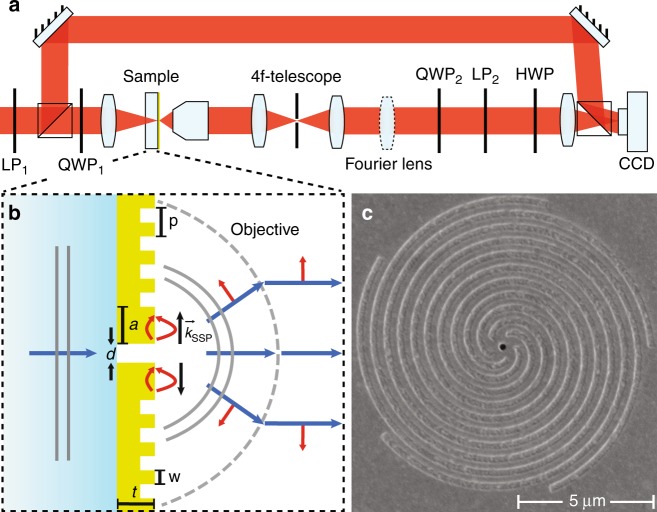
Schematic view of the setup and plasmonic spiral antenna. **a** Combined Fourier polarimetry and holography setup, consisting of a transmission microscope, two polarimeters and a reference beam. The microscope contains a 4f-telescope for spatial filtering of light from a single antenna. **b** Principle of plasmonic bullseye/spiral nanostructure. Light impinging from the glass side excites the single hole in a thick metal film. SPPs launched by the hole at the air side scatter out at the spiral corrugation. The red arrows denote the directions of the SPP and free-space electric fields. **c** SEM micrograph of a *m* = −5 spiral antenna

Our novel experimental technique for angle-resolved amplitude, polarization, and phase imaging of single nano-objects is based on a combination of Fourier microscopy, polarimetry, and digital holography. As Fig. 2a illustrates, the basis is a high-NA imaging microscope composed of a HeNe laser source (*λ* = 633 nm), a ×100 objective (Nikon, NA = 0.9), a 20 cm tube lens and a CCD camera. Additionally, a Fourier lens (*f* = 200 mm) is inserted such that its focal point (after being relayed by a 4f-telescope) lies in the back focal plane of the microscope objective, with the Fourier plane of the sample projected onto the CCD camera. This projection maps the in-plane wavevectors **k**_||_ of the scattered light with a high angular precision of ~0.5°over the full 70° opening angle of the objective^28,29^. Two Stokes polarimeters enable one to control and measure the full polarization state of incoming and outgoing light for each wave vector^30^, on the proviso that one accounts for the conversion of the spherical waves scattered from the nanostructures to paraxial cylindrical beams by the microscope objective. Next, this setup utilizes off-axis digital holography for phase resolution^31,32^ by mixing the object beam, which contains the wave front scattered by a single nanostructure, with a reference beam derived from the same laser. This technique has the advantage of operating in a single-shot manner by making use of single digitally Fourier-transformed camera images for phase resolution, as opposed to delay scanning. Although digital holographic microscopy was recently applied to nanophotonic structures^33–36^, these studies were mostly aimed at real space, i.e., sample plane, imaging. However, real-space images of nanoantennae have the drawback of being restricted by the diffraction limit and, therefore, typically cover just a few camera pixels. In contrast, Fourier-space holography of single nanoantennae generates a signal over an entire CCD chip, providing far richer information.

### Spiral nanostructures

We demonstrate the large potential of Fourier-space holography by applying it to single plasmonic bullseye and spiral nanostructures, which are expected to generate significant orbital angular momentum content and spin–orbit coupling signatures in their response^11,30,37,38^. The nanostructures consist of grooves milled with a focused ion beam around a central aperture, where the groove duty cycle is 50%, and the groove–groove spacing (pitch) is 500 nm, selected for operation near the wavelength of 633 nm. The structures are milled in a 200 nm optically thick gold film evaporated onto a glass coverslip; see Fig. 2b, c for a schematic and scanning electron micrograph. Throughout the text the handedness of the spirals is defined when looking along the wave propagation direction. Using this convention, *m* denotes the number of spiral grooves with *m* > 0 for clockwise (CW), *m* < 0 for counter-clockwise (CCW) spirals, and *m* = 0 for bullseye structures. While the single sub-wavelength aperture is a non-directional scatterer, the grooves are expected to generate directional outcoupling (beaming) of surface plasmon polaritons (SPPs) that are launched when exciting the aperture^4,39^. According to earlier reports^37,40^, these structures should imprint OAM onto scattered light, governed by the propagation delay experienced by the SPPs en route from the aperture to the outcoupling grooves and through a phenomenon called spin–orbit coupling^41–43^, which introduces changes between the incident and the outgoing spin angular momentum (SAM). For plane waves, SAM is associated with helicity so that *σ* = +1 and *σ* = −1 for right-hand circular (RHC) and left-hand circular (LHC) polarizations, respectively. Additionally, in this case, the term OAM does not refer to the common OAM studied for paraxial, cylindrical beams, but rather indicates that the scattered spherical wave ${\mathbf{S}}\left( {\theta ,\phi } \right)\frac{{e^{ikR}}}{R}$ carries helical phase fronts of the form exp(*ilϕ*) in its complex scattering amplitude function. Here, *l* is an integer and *ϕ* is the azimuthal angle, while we denote the angle relative to the optical axis through the gold film by *θ*.

### Experimental workflow

**Fig. 3:**
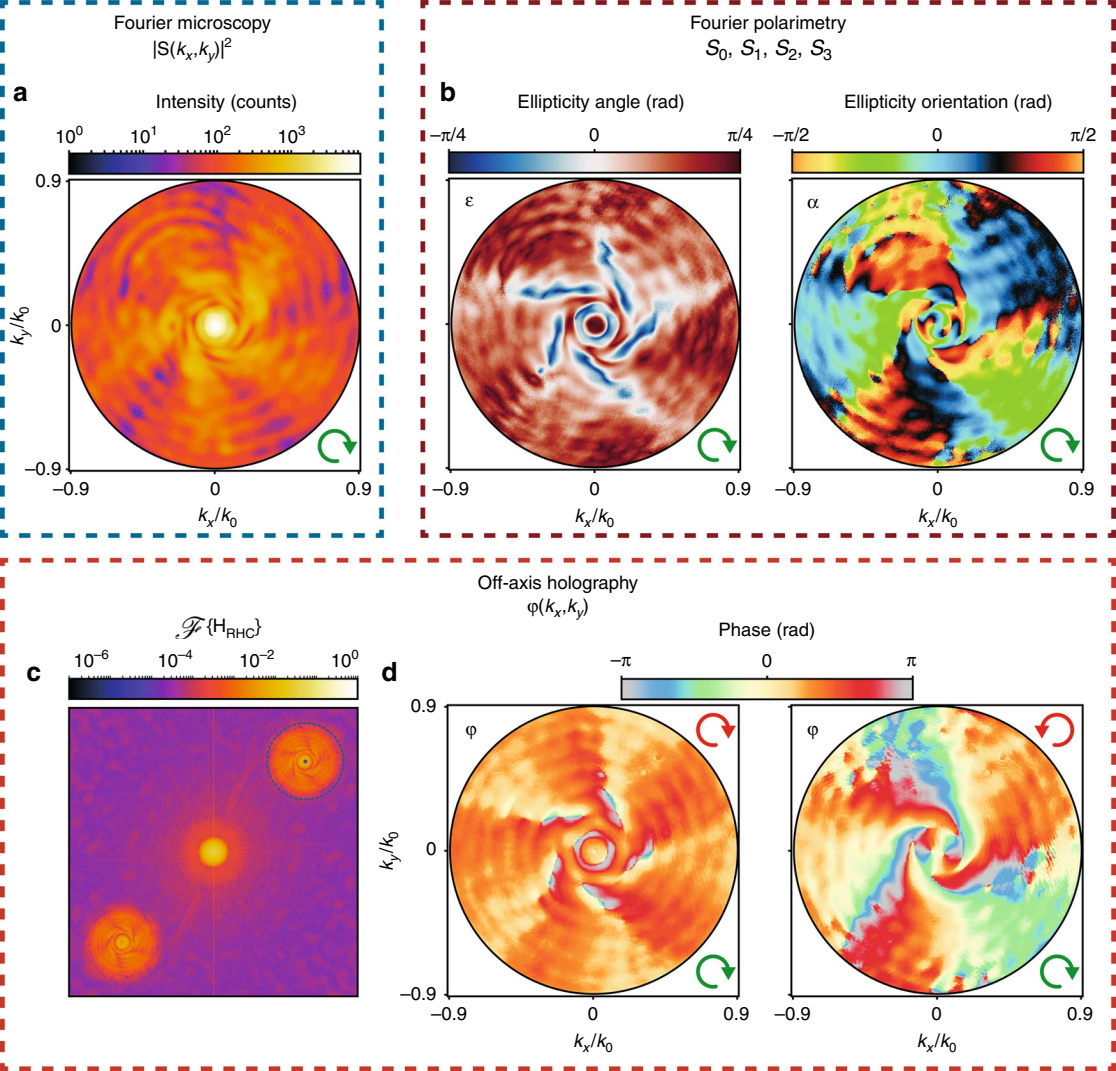
Demonstrations of Fourier microscopy, polarimetry, and holography. **a** Fourier map of intensity, **b** polarization ellipse parameters $\epsilon$ and *α*, **c** digital Fourier transform of an interferogram obtained with RHC polarized detection, **d** reconstructed phase profiles for RHC- and LHC-polarized detection. In all sub-figures, RHC polarized input and a *m* = −5 spiral nanostructure were used. The green and red arrows indicate the input and output polarizations, respectively

Figure 3 highlights the main measurement modalities required to fully quantify an antenna radiation pattern, taking a *m* = −5 spiral nanostructure with RHC excitation (input polarization indicated as green arrows in all figures) as an example. First and foremost, our setup is a Fourier microscope, which enables one to record the intensity radiation pattern |**S**(*θ*, *ϕ*))|^2^ or more precisely |**S**(*k*_*x*_, *k*_*y*_)|^2^d*k*_*x*_d*k*_*y*_ with (*k*_*x*_, *k*_*y*_) = (cos*ϕ*sin*θ*, sin*ϕ*sin*θ*). The intensity distribution in Fig. 3a (logarithmic scale) was measured by removing the polarization filters (QWP_2_, LP_2_, and HWP) in the collection path and blocking the reference beam in the setup shown in Fig. 2a. It features a high-intensity peak in its center, which signifies strong beaming into a narrow cone of angles normal to the sample. As shown in previous studies, this is a consequence of diffractive outcoupling of surface plasmon polaritons by the grooves^4,30,39^. This beaming stands in sharp contrast to an isolated hole, which would produce an isotropic pattern^30^. The |*m*| spiraling fringes and oscillations in the radial direction in the intensity map are caused by interference of the near-spherical wave scattered by the central aperture and the helically phase wave out-coupled by the spiral grooves^37^.

The second capability of the setup, namely, polarization-resolved imaging, relies on measurements of intensity profiles in different output polarization bases (linear along 0°, 45°, 90°, 135° and circular left and right, red arrows indicate polarization in each panel) to determine the Stokes parameters. An example raw data set with the corresponding Stokes parameter results is reported in the Supplementary. The Stokes parameters *S*_*i*_ with *i* = 0, 1, 2, 3 fully characterize the polarization state of the wavefront for each collected wave vector in the radiation pattern^44^ and can therefore be converted into a polarization ellipse for each detected wave vector in the radiation pattern (see methods section). Figure 3b reports the polarization ellipse parameters, namely, the ellipticity angle $\epsilon$ ($\epsilon$ = 0 for linear polarization, resp. ± *π*/4 for RHC/LHC polarization) and the orientation *α* of the major axis of the ellipse relative to the *k*_*x*_ axis. In the particular case of RHC input polarization, $\epsilon$ = −*π*/4 represents full helicity conversion, and $\epsilon$ = *π*/4 represents the retained polarization. The wavevector-resolved $\epsilon$ map in Fig. 3b) clearly shows complete helicity conversion ($\epsilon$ = −*π*/4) in a doughnut-like shape with five spiraling arms around it. Regions close to these features are characterized by an equal distribution of LHC and RHC polarization ($\epsilon$ = 0, linear polarization), with the remaining *k*-space containing an unchanged helicity ($\epsilon$ = *π*/4). This directly evidences helicity conversion at oblique scattering angles, i.e., spin–orbit coupling in scattering. The reader should note that since the nanostructure scatters the incident beam (cylindrical geometry) into a spherical wave, the polarization definition is not trivial. For instance, given that our input polarization RHC implies an input E-field vector along $\left( {\hat x + i\hat y} \right)/\sqrt 2$(with *z* the optical axis), the definition of the helicity conserving/reversing channel actually means projection of the full measured field **S** on $\left( {\hat \theta \pm i\hat \phi } \right)/\sqrt 2 .$

Finally, while the Stokes parameter measurements allow for the retrieval of phase differences between two orthogonally polarized field components, by adding digital holographic microscopy capabilities to the setup, we are able to measure the individual phase profiles of the respective polarization states. More specifically, we make use of digital off-axis holography. To this end, the reference beam *E*_*R*_, polarized identical to the detection channel, is overlapped with the object wave $E_O = \left| {E_O} \right|\exp \left[ { - i\varphi } \right]$, i.e., the Fourier-space image, on the CCD camera, which results in the hologram *H* = |*E*_*O*_ + *E*_*R*_|^2^. Considering the simplest case of the reference beam being a plane wave with $E_R = \left| {E_R} \right|\exp \left[ { - i\vec k \cdot \vec x\,{\mathrm {sin}}\delta } \right]$, where $\vec k$ is its wavevector and *δ* the angle between the object and reference beam, the hologram *H* can be mathematically expressed as

1 $$H = \left| {E_O} \right|^2 + \left| {E_R} \right|^2 + \left| {E_O \cdot E_R} \right|\exp \left[ { \pm i\left( {\varphi + \vec k \cdot \vec x\,{\mathrm {sin}}\delta } \right)} \right]$$

From this equation, it becomes apparent that only the cross terms contain *φ*, which is the object wave phase information. The additional phase term $\vec k \cdot \vec x\,{\mathrm {sin}}\delta$ arises from the deliberately introduced tilt *δ*, which serves to separate the cross and direct terms upon digital Fourier transformation. Figure 3c shows such a digital Fourier transform of the hologram corresponding to a *m* = −5 spiral in circular co-polarization. This inclination angle *δ* is chosen to be sufficiently large such that upon Fourier transformation, the cross and direct terms are well separated yet sufficiently small that the hologram fringes are well sampled by the pixelated detector. The remaining digital reconstruction process consists of selecting (i.e., binary masking), shifting, back-Fourier transforming one of the cross-terms and a digital correction for residual parabolic phase aberrations in the imaging optics (see Methods section). Figure 3d shows two measured phase maps for the co- and cross-polarized channel. They reveal a helical evolution of the phase around the optical axis, which is especially evident for the cross-polarized channel. The phase profile in the co-polarized channel contains five spiraling arms, with an overall phase increment of 5⋅2*π* when going full circle around the origin. The phase profile in the cross-polarized channel contains 2 fewer arms and an additional feature around its center. The origin of these effects will be further discussed in the next section.

### Analysis of spiral radiation patterns

**Fig. 4:**
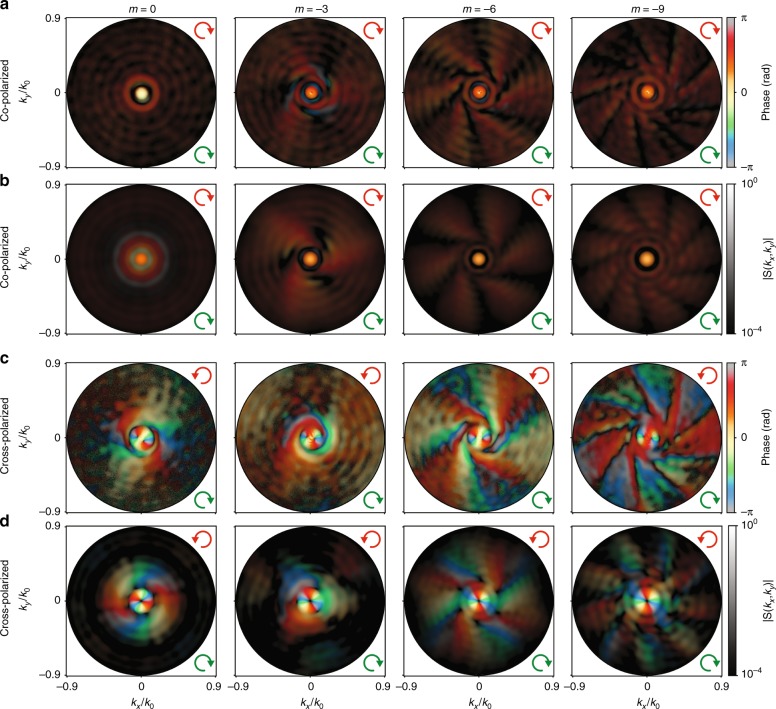
Example set of interferometric and polarimetric k-space microscopy measurement and simulation results. **a**, **c** Measured and **b**, **d** simulated complex field profiles in the Fourier plane of a bullseye (*m* = 0) and *m* = −3, −6, −9 spirals. The transmitted polarization channels are co-polarized (**a**, **b**) and cross-polarized (**c**, **d**) with RHC-polarized input. The plots show a combined representation for the phase as hue and field amplitude as brightness (logarithmic scale). The green and red arrows indicate the input and output polarizations, respectively. Each amplitude profile is normalized by its maximum. A circular representation of the color map used here is given in Fig. S3 **c**

Having introduced the experimental workflow for full quantification of the amplitude, polarization and phase information in the radiation of a nano-object, we present an example of the type of rich insight one can gain. For plasmonic spirals, we conducted an in-depth quantification of their OAM-conversion efficiency depending on their geometry, i.e., on the number of arms. Figure 4a, c demonstrates a subset of the measured full electric fields radiated into the polarization-helicity-conserving and helicity-changing channel using RHC input polarization. To support our measured results, we show the simulated field profiles of the corresponding spiral/bullseye structures in Fig. 4b, d, which demonstrate a good qualitative agreement. These plots show simultaneously the field phase (from hologram) as hue and the field amplitude (|***S***(*θ*, *ϕ*)|) as brightness (logarithmic scale). Figure 4 highlights results for CCW spirals with up to 9 arms (*m* = −9), starting from the case of a bullseye (*m* = 0). It is evident that the number of spiral grooves |*m*| has a big impact on both the amplitude and phase in both co-polarized and cross-polarized measurements. As in Fig. 3d, the number of spiraling arms in the Fourier maps coincides with the number of arms of the spirals in co-polarization, while in cross-polarization, the field profiles show 2 fewer arms. These arms are visible both in the amplitude and phase because they bring a phase increment of 2*π* per arm when traversing a circle at constant *θ* through the radiation pattern. These observations highlight that OAM conversion is controlled by a combination of propagation phase (number of arms) and SAM conversion (±2 for helicity-reversing scattering). We refer to Fig. S4 in the Supplementary for further examples with the same input/output polarization settings but with a reversed spiral orientation (*m* > 0). In this case, not only does the orientation of the field profiles flip but also the observed number of arms in cross-polarization is increased, instead of diminished, by an offset of 2 compared to the number of physical grooves. In all datasets, the overall pattern is quite different near the center of the images, i.e., for radiation angles near-normal to the gold film. All co-polarized radiation patterns show a bright feature exactly at *k*_||_ = 0 with a flat phase, while the cross-polarized measurements reveal a donut-shaped feature with a phase advance of 2⋅2*π* when traversing a circle around the optical axis. The OAM transfer corresponding to this donut-shaped feature is equal to the change in the SAM (Δσ = ±2), independent of the geometry or handedness of the grooves. Since this feature persists in the case of a bullseye geometry, we conclude that it arises from light that has undergone spin-to-orbital-angular-momentum conversion caused by the subwavelength aperture and scattered in the normal direction (*k*_||_ = 0) into the far field^44^. This is confirmed at the end of this section by OAM decomposition of the measured fields. In any case, we conclude that the radiation patterns do not map directly onto the well-known OAM-carrying Laguerre–Gauss beams. Instead, the spiral structures scatter into a variety of OAM contributions, the superposition of which is observed as a radiation pattern.

The observations indicate that the conversion of input plane waves into output OAM-carrying spherical waves leads to an OAM mixture, in contrast to the “binary” selection rules reported earlier^37^. The expected selection rules originate from a simple picture, taking the central aperture as a point launching SPPs that then accumulate propagation delay due to the twist of the spiral grooves, where light is coupled into free space. The resulting superposition of wavefronts at an *m*-armed spiral results in an offset of *m* in the OAM index. Spin-to-orbital angular momentum conversion causes an additional offset of ±2 in the generated OAM mode for helicity-non-conserving detection^41–43^. This leads to the selection rule:

2 $$\Delta l = m + \Delta \sigma$$

which states that the amount of generated OAM mode difference Δ*l* compared to the input wave (OAM = 0 in this work) should be equal to the number of spiral arms *m* plus the helicity change Δ*σ* = 0, ±2 (conserving, resp. conversion from RHC to LHC or vice versa)^37^.

A main strength of our measurement scheme is that the purity of the selection rule can be quantitatively analyzed by computationally decomposing the complex field into the desired basis. This is done by representing the complex electric field *E*(*θ*,*ϕ*) as a linear combination of OAM states:

3 $$E\left( {\theta ,\phi } \right) = \mathop {\sum}\limits_{l = - \infty }^\infty {C_l\left( \theta \right)} \exp \left( {il\phi } \right)$$

where, to take into account the spherical nature of the scattered wave, we use spherical coordinates, with coefficients *C*_*l*_ (*θ*) defined as

4 $$C_l\left( \theta \right) = \frac{1}{{2\pi }}\mathop {\int}\limits_0^{2\pi } {E\left( {\theta ,\phi } \right).\exp \left( { - il\phi } \right){\mathrm{d}}\phi}$$

The complex-valued, azimuthal overlap integral results *C*_*l*_(*θ*) can be used to calculate the polar angle resolved OAM mode density (see Fig. S5 a, b). From this, the purity or power of an OAM mode *l* is derived as

5 $$p_l = \mathop {\int}\limits_0^{\arcsin \left( {NA} \right)} {\left| {C_l\left( \theta \right)|^2} \right|} .\sin \left( \theta \right){\mathrm d}\theta$$

Normalizing *p*_*l*_ by the sum of its values results in the modal power spectrum $P_l = p_l/\mathop {\sum }\limits_{l = - \infty }^\infty p_l$ (see Supplementary for implementation details).

**Fig. 5:**
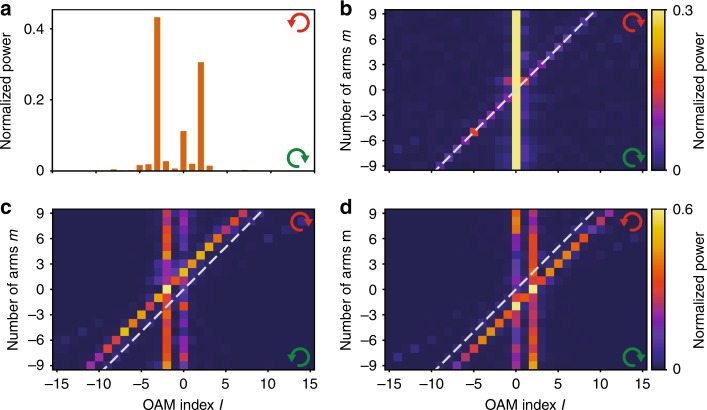
OAM decomposition results. **a** OAM power spectrum for a *m* = −5 spiral with RHC input and LHC output. **b**–**d** OAM power spectra as a function of *m* in co-polarization (**b**) and cross-polarization (**c**, **d**) with RHC (**b**, **d**) and LHC (**c**) polarized input. The dashed lines in **b**–**d** indicate *l* = *m*. Sub-figures **c** and **d** have the same color scale

As an example, Fig. 5a reports the OAM power spectrum for a *m* = −5 spiral in the helicity-non-conserving channel with RHC input, showing dominant contributions at Δ*l* = + 2, Δ*l* = 0 and Δ*l* = −3. Figure 5b–d combines the OAM mode decomposition results for *m* = −9 to *m* = 9 spirals (each OAM power spectrum is normalized to unit integrated content) for three different input–output polarization combinations. Throughout we find dominant features at the selection rule^37^, i.e., at the diagonal *l* = *m* in the helicity-conserving channels (Fig. 5b and Fig. S5 c), *l* = *m* ∓ 2, respectively, for conversion from LHC to RHC (Fig. 5c) and vice versa (Fig. 5d). In addition, we observe strong leakage into the *l* = 0 mode for polarization-conserving measurement, resp. *l* = ± 2 for the polarization non-conserving data. On average, we identify the purities of the OAM modes following the selection rule to be (13 ± 4)% for co-polarization and (38 ± 3)% for cross-polarization.

In general, the dominant OAM contributions fit the interpretation that only a fraction of the light interacted with the spiral grooves, while the remainder essentially gained no OAM upon direct transmission or gained only OAM corresponding to a change of the SAM, i.e., spin–orbit coupling at the aperture. Additionally, the presence of the OAM mode *l* = 0 in cross-polarization is likely due to the non-perfect extinction ratio of the polarizers employed. While from the perspective of functional nanostructures for generating OAM, it is evident that performance is imperfect, these measurements highlight that our powerful new measurement method allows for an unprecedented quantification. The performance of the nanostructures in terms of OAM mode purity can be further improved by optimizing the geometrical parameters of the spiral such as the aperture radius or by using metal–insulator–metal arrangements^45,46^. Our observation points out that the essential parameter to optimize is the tradeoff between overall transmission efficiency (requires large central aperture) and leakage into OAM = 0, ±2 (requires low direct transmission). Detailed pointers for observation are available in the data as one has the full radiated field at hand. For instance, one can precisely determine the OAM mode density within any given band of angles *θ*, *θ* + Δ*θ*, enabling one to determine the polar angles into which particular OAM content is scattered (see Supplementary).

### Method validation and minimum redundancy

Returning to our measurement scheme, we note that polarimetry plus interferometry provides not only abundant but also redundant information on the wavefront. The reason for this lies in the fact that polarimetry already determines the phase difference between orthogonally polarized field components, which should be strictly identical to the difference in phase profiles measured with holography in the same two crossed polarizations. For instance, the following simple combinations of Stokes parameters represent phase difference profiles

6a $$\arg \left( {S_2 + iS_3} \right) = \varphi _V - \varphi _H$$

6b $$\arg \left( {S_1 - iS_3} \right) = \varphi _A - \varphi _D$$

6c $$\arg \left( {S_1 + iS_2} \right) = \varphi _{\mathrm {LHC}} - \varphi _{\mathrm {RHC}}$$

where *φ* is the phase profile corresponding to the polarization state labeled in the subscript (V = vertical, H = horizontal, A = anti-diagonal, D = diagonal, taken to refer to the camera plane). On one hand, this redundancy can be used to check the consistency of the method by comparing maps of phase differences determined with polarimetry and holography. On the other hand, one can use the redundancy to reduce the total number of measurements required to fully determine **S**(*θ*, *ϕ*) (up to an arbitrary phase offset).

**Fig. 6:**
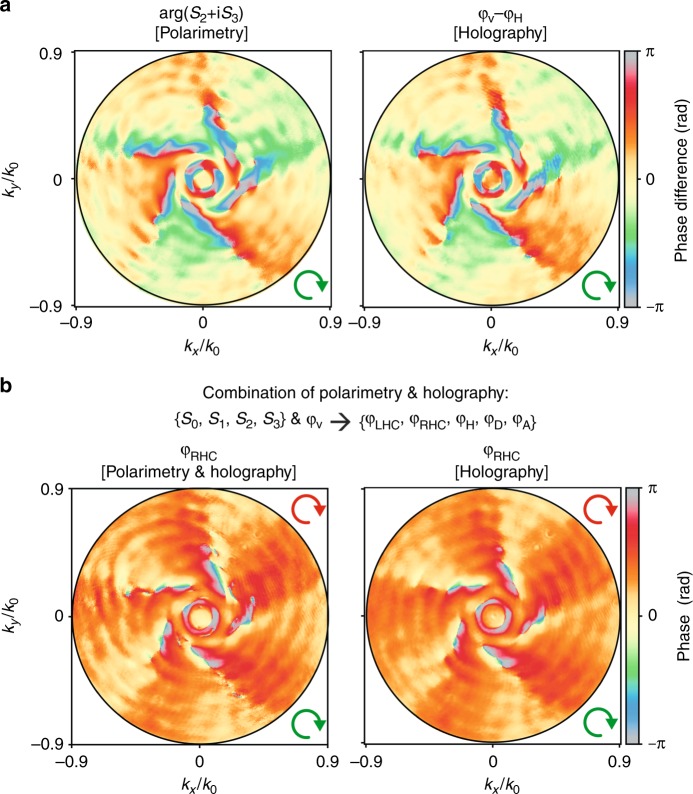
Validation and redundancy removal in combined holographic and polarimetric measurements. **a** Difference between phase profiles with vertical and horizontal output polarizations retrieved using polarimetry and holography. **b** Example of a phase profile with RHC polarized output, which was retrieved using a combination of polarimetry and holography, with the correspondent purely holographic profile shown for comparison. In both cases, a RHC-polarized input and a *m* = −5 spiral were used. The green and red arrows indicate respectively the input and output polarizations

Figure 6a shows an example of consistency check through a direct comparison of *φ*_*V*_−*φ*_*H*_ and arg(*S*_2_ + *iS*_3_) again for the case of a *m* = −5 spiral (the remaining two phase difference comparisons listed in Eq. 6 are shown in Fig. S6). Evidently, these measurements confirm the consistency between phase difference maps obtained using holography and polarimetry, with the only notable distinction lying in the noise characteristics. Stokes polarimetry relies on pixel-by-pixel image subtraction, leading to uncorrelated shot-noise propagating into the phase map. Instead, the holography images are effectively low-pass filtered by the reconstruction procedure (Fourier transform and masking of interferogram).

Figure 6b) provides an example of how the Stokes parameters in combination with a single holography measurement (in this case *φ*_*V*_) can be used to reconstruct the phase profile in any arbitrary polarization channel. Here, Fig. 6b shows the reconstructed phase profile for a *m* = −5 spiral for RHC-polarized detection, alongside a direct holographic determination of the phase for comparison. This illustrates how the number of measurements needed to fully quantify a radiation pattern can be reduced. Instead of independent polarimetry and holography measurements for each polarization, it is enough to perform a single holographic measurement plus the four measurements required to retrieve the Stokes parameters (required calculus is described in the Supplementary). In addition, the phase profile reconstructions for four additional polarization channels (vertical, diagonal, anti-diagonal, LHC) are shown in Fig. S6. These comparisons show excellent agreement.

The minimally redundant measurements could be advantageous both from the viewpoint of efficiency as well as reduced requirements on camera dynamic range in polarimetry compared to holographic measurements. Holography works best with a large fringe contrast throughout the recorded interferogram, with fringe intensities spanning the camera dynamic range. This fringe contrast (visibility) increases as the intensity ratio of the two interfering waves (object and reference wave) gets closer to one^31^. For strongly structured radiation patterns, this requirement is difficult to fulfill. The data redundancy enables one to perform the holography just for a polarization channel that has the most suited intensity distribution, i.e., polarization channels where directly transmitted light is (partially) blocked. On top of that, this approach allows for improvements in measurement speed, since the minimum number of required images can be reduced to only four: a single hologram to determine amplitude and phase in one polarization channel, and intensity measurements through three complementary polarizer settings.

## Discussion

We developed a measurement technique for the characterization of scattered radiation patterns from individual nanoscatters in terms of amplitude, vector, and phase. In principle, the technique could be performed even with a single camera shot, given that off-axis holography requires just a single image and that polarization can be multiplexed^47^. As an example application demonstrating the remarkable insight that this technique can offer for studies of optical antennae, we analyzed the OAM content of light scattered by a family of plasmonic spirals. In contrast, with other OAM measurement techniques, our method does not require the use of spiral phase plates or holograms^23,24^, with a single image enabling direct decomposition in OAM contributions. The current limit to our technique is the NA of our microscope objective. Extending the method to a 4*π* microscopy arrangement could fulfill the quest for full quantification of the multipole content of any scattering geometry using its far field. The presented method will directly apply to many important problems in nanophotonics, such as the use of plasmonic oligomer antennae for sensing and emission, metasurfaces for controlling transmitted and reflected wavefronts that depend on the incident amplitude, phase and vector contents, and nonlinear metasurfaces in which phase gradients imprinted in the metasurface geometry determine the efficiency and angular distribution of, e.g., frequency conversion. In all these cases, access to angle-resolved amplitude, polarization and phase properties of the far-field is of paramount importance.

## Materials and methods

### Experimental setup

The laser beam (frequency-stabilized Helium Neon laser *λ* = 633 nm) is expanded by a telescope (*f* = −25 mm and *f* = 200 mm achromats) and transmitted through the first polarimeter consisting of a linear polarizer LP_1_ (Thorlabs LPVIS100) and quarter wave plate QWP_1_ (Thorlabs AQWP05M-600), which are used to prepare the incoming polarization state. After LP_1_, the beam is split in two by a 50/50 beamsplitter. In the object arm, the beam is weakly focused (*f* = 125 mm) onto the center of a nanostructure. Light scattered by the nanostructure is collected from the patterned gold side by a Nikon LU Plan Apo ×100/0.9 objective. The scattered light is guided through a 4f-telescope (two *f*_*T*_ = 50 mm lenses), which contains a circular pinhole (diameter of 400 µm) as a real space filter to select only the light coming from the nanostructure. The Fourier lens (*f*_*F*_ = 200 mm) is placed at a distance 4*f*_*T*_ + *f*_*F*_ from the objective back focal plane, just behind the telescope. A second polarimeter consisting of a quarter wave plate QWP_2_ (Thorlabs AQWP05M-600) and linear polarizer LP_2_ (Thorlabs LPVIS100) projects the outgoing wave onto the desired polarization channel. A half waveplate (Thorlabs AHWP05M-600) is included to rotate the polarization of the object wave back onto that of the reference wave, which is recombined with the object wave using a second 50/50 beamsplitter. The tube lens (*f* = 200 mm) creates a real (Fourier) space image on the CCD (Photometrics CoolSNAP EZ) in the absence (presence) of the Fourier lens that resides on a flip mount. To increase the dynamic range of our measurement beyond that of the CCD camera, we record the intensity and interference patterns using two different exposure times (*t*_1_ = 50 ms and *t*_2_ = 2000 ms) and merge the two images by using *I*(*t*_1_) in a circular region around the high-intensity part of the image and *I*(*t*_2_) for the remainder, while compensating for the ratio of integration times. The reference beam is attenuated by optical density filters to approximately half of the intensity maximum of the object wave in the co-polarized channel.

### Sample design and nanofabrication

Samples were fabricated on 170 µm thick glass cover slides with a 5-nm-thick chromium adhesion layer and a 200-nm-thick gold film evaporated on top. Structures were milled into the gold film using a focused ion beam (FEI Helios). The geometrical parameters are: central aperture diameter *d* = 250 nm, separation of the first groove and the center *a* = 375 nm, width of grooves *w* = 250 nm, pitch of grooves *p* = 500 nm, and number of windings *N* = 10. The central aperture is milled entirely through the gold and chromium, while the corrugations are ~80 nm deep. While for the bullseye structures, these corrugations are concentric, for the *m*-armed spirals (*m* > 0 for CW and *m* < 0 for CCW spirals) the radius *r* depends on the azimuthal angle *ϕ* according to:

7 $$r\left( \phi \right) = a + m \cdot p\frac{\phi }{{2\pi }}0 \le \phi < 2\pi N$$

In the case of |*m*| > 1, adjacent spiral arms have an angle offset of Δ*ϕ* = 2*π*/|*m*|. The minimal separation between neighboring spirals is 25 µm to prevent inter-structure coupling.

### Stokes and polarization ellipse parameters

The Stokes parameters *S*_*i*_, where *i* = 0, 1, 2, 3, can be written in terms of orthogonal electric field components *E*_*x*_ and *E*_*y*_ as

8 $$\left( {\begin{array}{*{20}{c}} {S_0} \\ {S_1} \\ {S_2} \\ {S_3} \end{array}} \right) = \left( {\begin{array}{*{20}{l}} {\left\langle {\left| {E_x} \right|^2} \right\rangle } \hfill & + \hfill & {\left\langle {\left| {E_y} \right|^2} \right\rangle } \hfill \\ {\left\langle {\left| {E_x} \right|^2} \right\rangle } \hfill & - \hfill & {\left\langle {\left| {E_y} \right|^2} \right\rangle } \hfill \\ {\left\langle {E_xE_y^ \ast } \right\rangle } \hfill & + \hfill & {\left\langle {E_yE_x^ \ast } \right\rangle } \hfill \\ {i\left( {\left\langle {E_xE_y^ \ast } \right\rangle } \right.} \hfill & - \hfill & {\left. {\left\langle {E_yE_x^ \ast } \right\rangle } \right)} \hfill \end{array}} \right) = \left( {\begin{array}{*{20}{c}} {I(0^\circ ,0^\circ )} & + & {I(90^\circ ,90^\circ )} \\ {I(0^\circ ,0^\circ )} & - & {I(90^\circ ,90^\circ )} \\ {I(45^\circ ,45^\circ )} & - & {I(135^\circ ,135^\circ )} \\ {I(0^\circ ,45^\circ )} & - & {I(0^\circ ,135^\circ )} \end{array}} \right)$$

Here, *x* and *y* refer to camera coordinates. We collect the six intensity maps *I*(*β*, *γ*), i.e., *I*_*H*_, *I*_*V*_, *I*_*D*_, *I*_*A*_,*I*_RHC_, *I*_LHC_ with the required angles *β* and *γ* for QWP_2_ and LP_2_ indicated in Eq. 8. It should be noted that this set is redundant, as in principle four measurements suffice (e.g., *I*_*H*_, *I*_*V*_, *I*_*D*_, *I*_RHC_). The polarization ellipse parameters (sketch shown in Fig. S2a) can be derived from the Stokes parameters using

9a $${\epsilon} = \frac{1}{2} \cdot \arg \left( {\sqrt {S_1^2 + S_2^2} + iS_3} \right)$$

9b $$\alpha = \frac{1}{2} \cdot \arg \left( {S_1 + iS_2} \right)$$

### Phase correction in off-axis holography

In digital holographic microscopy phase aberrations can occur due to the high NA microscope objective and a non-flat phase front for the reference beam^32^. The aberration consists of an overall parabolic phase profile that is present already when imaging a single subwavelength aperture. We perform a numerical correction using the complex conjugate of the spherical phase term that results in the flattest residual when multiplied with the phase measured for a single aperture^32^. This phase correction calibration step needs to be repeated each time the sample position, objective focus or reference beam tilt angle change. Note, that although the curvature of the parabolic phase correction value does not influence the retrieved OAM power spectrum, lateral misalignments of the phase correction with respect to the propagation axes can cause a minor mode crosstalk.

### Simulation procedure

Far-field profile simulations are performed using 3D finite-difference time-domain software (Lumerical FDTD). The bullseye/spiral structures are modeled using the same parameters as the fabricated structures and are excited using two normally incident plane waves, which are shifted 90° in phase and rotated 90° in polarization (RHC polarized input) and have a wavelength of λ = 633 nm. The total simulation region has dimensions (17 × 17 × 0.8)µm³ and is enclosed by perfectly matched layers (PMLs). A mesh grid size of 12 nm is used to model the spiral grooves. The gold film permittivity was modeled using a Drude model fit to values reported by Johnson and Christy:^48^

10 $${\it{\epsilon }}_r = {\it{\epsilon }}_\infty - \frac{{\omega _p^2}}{{\omega \left( {\omega + i\gamma } \right)}}$$

where $\epsilon$_∞_ = 9.54, *ω*_*p*_ = 1.35 × 10^16^ rad/s and *γ* = 1.25 × 10^14^ rad/s. The electromagnetic field is recorded using a monitor on a plane located 10 nm above the nanoscatterer; the standard Lumerical Stratton–Chu near to the far-field projection technique is applied to calculate the field 1 m away from the structure. It should be noted that this technique is not rigorous for systems with interfaces.

## Electronic supplementary material

Supplemental Material
