# Supplementary material for: Quantifying single plasmonic nanostructure far-fields with interferometric and polarimetric k-space microscopy
Source: Light Sci Appl. 2018 Sep 12;7:65. doi: 10.1038/s41377-018-0059-0 (PMC6134066; doi:10.1038/s41377-018-0059-0)
Supplement: Supplementary file 1 — Supplemental Material [file 41377_2018_59_MOESM1_ESM.pdf]

Supplementary Information for: Quantifying single plasmonic nanostructure far-fields with interferometric and polarimetric k-space microscopy

Ruslan Röhrich[1,2], Chris Hoekmeijer[1], Clara I. Osorio[1], A. Femius Koenderink[1*]

1. Center for Nanophotonics, AMOLF, Science Park 104, 1098 XG Amsterdam, The Netherlands
2. ARCNL, Science Park 110, 1098 XG Amsterdam
*Corresponding author: f.koenderink@amolf.nl

I Description of Stokes polarimetry

Stokes polarimetry is a technique that can be used to completely characterize the polarization state of light, which includes partially polarized light, by using four measurable quantities called Stokes parameters [1]. Experimentally these quantities can be retrieved through six intensity measurements, each projected in the appropriate polarization channel. The necessary settings for the quarter-wave plate and linear polarizer in the polarimeter are described in the methods part of the paper. An example set of such intensity measurements with six different outgoing beam polarizations (horizontal, vertical, diagonal, anti-diagonal, LHC and RHC) for the $m = -5$ spiral and RHC polarized input is shown in Fig. S 1 a). Simulation results for corresponding intensity profiles are shown Fig. S 1 b). In addition to intensities, we recorded interference patterns in each of the six different polarization channels and retrieved phase profiles using off-axis holography, which are presented in Fig. S 1 c). For comparison, simulated phase profiles in corresponding polarization channels are shown in Fig. S 1 d).
Using the set of intensity measurements in Fig. S 1 a) the Stokes parameters shown in Fig. S 2 were calculated. Here the Stokes parameters S_i, $i = 1, 2, 3$ are normalized by S_0 and therefore range from -1 to $+1$. The physical connotation of the Stokes parameters can be described as follows:

- S_0 is equivalent to the total field intensity.
- S_1/S_0 quantifies whether the light is preferentially linearly polarized in the horizontal/vertical basis (positive, resp. negative values).
- S_2/S_0 quantifies whether the light is preferentially linearly polarized in the diagonal/antidiagonal basis (positive, resp. negative values).
- S_3/S_0 describes the degree of circular polarization (sign encoding for helicity).
- Additionally, $\sqrt{\mathrm{S}_1^2 + \mathrm{S}_2^2 + \mathrm{S}_3^2}/\mathrm{S}_0$ describes the degree of polarization.

Please note that while the field intensity $|S(k_x, k_y)|^2$ shown in Fig. 3 a) is the same physical quantity as the Stokes parameter S_0 shown in Fig. S 2, operationally S_0 was determined by summing polarization channels, averaging over three measurement basis choices, i.e. $S_0 = (I_H + I_V + I_D + I_A + I_{RHC} + I_{LHC})/3$. The minor differences between the two plots are explained by a higher signal to noise ratio of the latter approach.
Fig. 3 a) shows a sketch of the polarization ellipse, which was used in the main text to illustrate typical polarimetry measurement results. The relationship between the polarization ellipse parameters and the Stokes parameters is given in the methods part of the paper. From Fig. 3 a) it becomes clear that the polarization becomes circular in case of $\epsilon = \pm\pi/4$ and linear for $\epsilon = 0$.

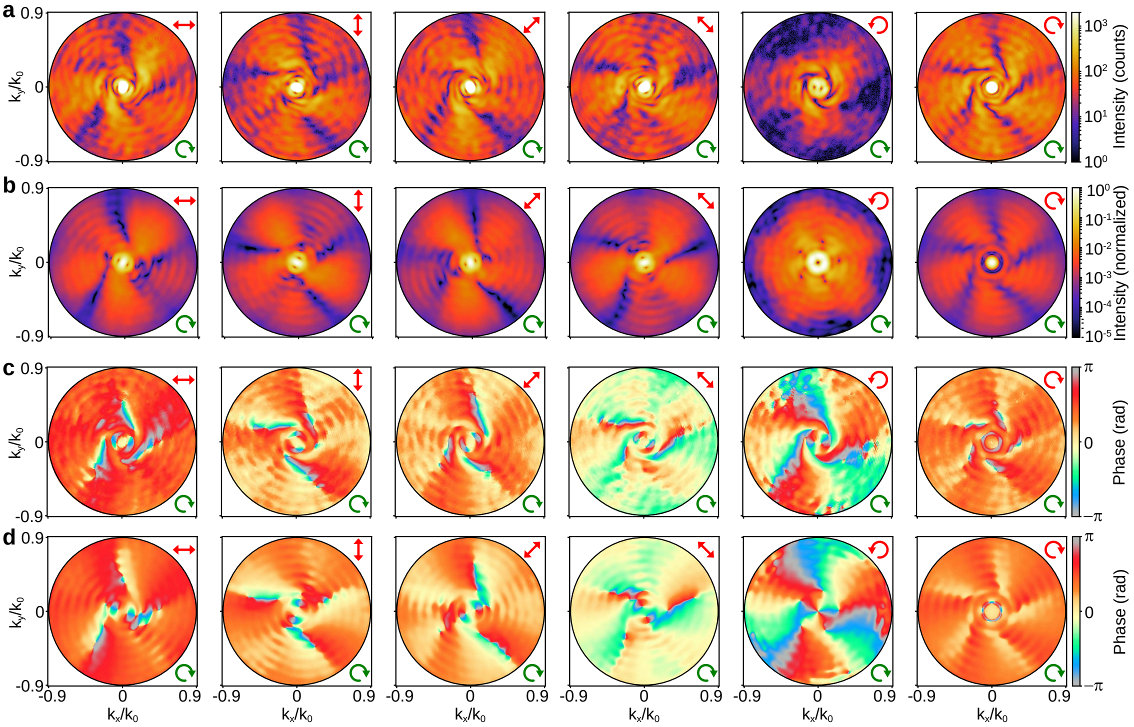

Figure 1. Example set for intensity and phase profiles. (**a-b**) Measured and simulated intensity profiles in units of CCD counts and normalized units respectively, for a $m = -5$ spiral for 6 different outgoing polarizations and RHC polarized input. (**c-d**) Phase profiles from holography measurements and from simulations for the same structure and polarization channels. The green and red arrows indicate the input and output polarizations, respectively.

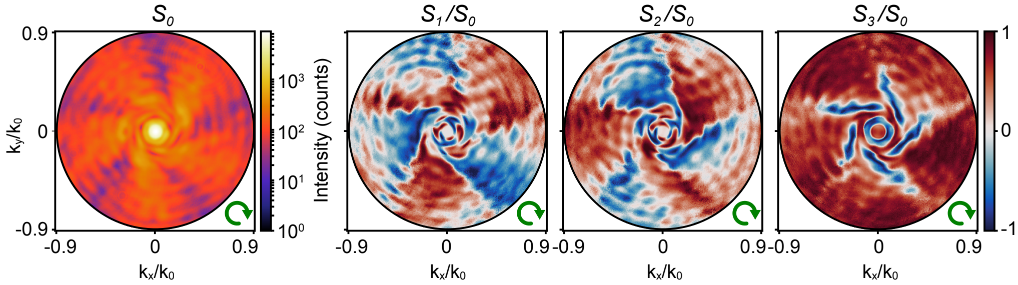

Figure 2. Example set of Stokes parameters. Stokes parameter results for a $m = -5$ spiral with RHC polarized input.

II OAM decomposition procedure

In the following, we describe the implementation of the OAM decomposition method. As mentioned in the main text, we make use of the measured complex field profile by computationally decomposing the field into the fundamental helical modes $\exp(il\phi)$. Since we are using a CCD camera, the measured field $E_{obs}(x, y)$ is discretized and given in Cartesian coordinates. To take this into account, Eqs. 4 and 5 in the main article need to be transformed into Cartesian coordinates. This can be achieved by substituting the coordinates and including the appropriate Jacobian. The Jacobian for the spherical to Cartesian coordinates transformation can be calculated using the inverse of the two Jacobian determinants given in Table S 1 and is given by:

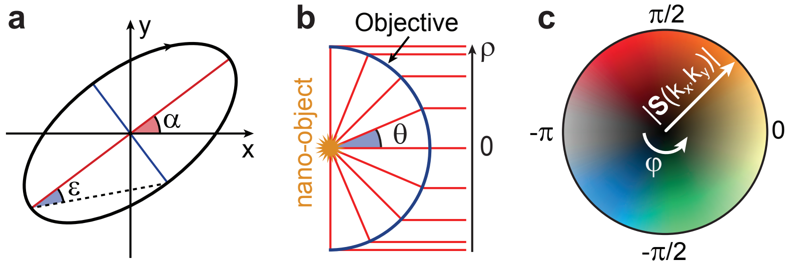

Figure 3. Polarization ellipse, apodization and circular color map. (**a**) Polarization ellipse representation of an elliptically polarized wave showing the parameters α and ϵ. (**b**) Illustration of the apodization effect occurring in high NA objectives. A point source radiates an isotropic, spheric wave, which is turned into a collimated beam with a non-uniform intensity distribution in the back-focal plane of the objective. The increased ray density at higher ρ indicates the higher intensities. (**c**) A circular representation of the color map used to jointly represent amplitude and phase in Fig. 4 and Fig. S 4.

$$J = \frac{1}{f\cos\theta} \cdot \frac{1}{\rho}, \tag{1}$$

where f is the objective focal length, $\cos(\theta) = \sqrt{1-\rho^2/f^2}$ and $\rho = \sqrt{x^2+y^2}$. By using this Jacobi determinant, substituting spherical coordinates and restricting the integration region to $\rho = f\sin(\theta)$, the integral in Eq. 4 from the main text can be written as:

$$C_l(\theta) = \frac{1}{2\pi} \iint_{\rho = f\,\sin(\theta)} E(x,y) \cdot \exp\left(-il\phi\right) \cdot \frac{1}{f\,\cos(\theta)\rho}\,\mathrm{d}x\,\mathrm{d}y, \tag{2}$$

where $\phi = \arctan\left(y/x\right)$. In practice the integral in Eq. 2 is replaced by a discretized sum over x and y coordinates, while a ring-shaped, binary mask

$$R_\theta(x,y) = \begin{cases} 1 & \text{for} \quad \theta - \Delta\theta/2 < \arcsin\left(\frac{\sqrt{x^2+y^2}}{f}\right) < \theta + \Delta\theta/2 \\ 0 & \text{else.} \end{cases} \tag{3}$$

is introduced as a factor in the integrand to confine the integral to a narrow, annular region, whereby the angle θ is varying in a range of $\theta = 0$ and $\theta = \arcsin(\mathrm{NA})$. For infinitesimally small $\Delta\theta$ this procedure becomes equivalent to the integration over ϕ described in Eq. 4 in the main text. In this work, we split the θ range in 100 parts to get accurately resolved $C_l(\theta)$ overlap integrals.

Furthermore, when using the Fourier imaging technique for quantitative purposes, a general property of high NA objectives called apodization needs to be considered. This purely geometric effect causes isotropic spherical waves to be transformed into collimated beams with non-uniform intensity profiles, since the objective lens transformation causes increasing ray densities at increasing polar angles θ, as shown in Fig. S 3 b) [2, 3]. Given that our objective satisfies Abbe's sine condition, this effect leads to the following relation between the electric field E and the observed electric field E_{obs}: $E = E_{obs} \cdot \sqrt{\cos(\theta)}$. This results

Cylindrical to spherical	Cartesian to cylindrical
$\det\left\{\frac{\partial(\rho,\phi)}{\partial(\theta,\phi)}\right\} = \det\begin{pmatrix} f\,\cos(\theta) & 0 \\ 0 & 1 \end{pmatrix} = f\,\cos(\theta)$	$\det\left\{\frac{\partial(x,y)}{\partial(\rho,\phi)}\right\} = \det\begin{pmatrix} \cos(\phi) & -\rho\sin(\phi) \\ \sin(\phi) & \rho\cos(\phi) \end{pmatrix} = \rho$

Table S 1. Jacobian determinants for cylindrical to spherical and Cartesian to cylindrical transformations.

in the following modification of Eq. 2:

$$C_l(\theta) = \frac{1}{2\pi} \iint_{\rho = f\sin(\theta)} \underbrace{\frac{E(x,y)}{\sqrt{\cos(\theta)}}}_{E_{obs}} \cdot \exp\left(-il\phi\right) \cdot \frac{1}{f\sqrt{\cos(\theta)}\rho} \,\mathrm{d}x\,\mathrm{d}y. \tag{4}$$

The OAM mode decomposition is performed for a finite range of OAM indices $l = -20...20$, whereby in the OAM power spectrum plots the index range was reduced to $l = -15,...,15$, where most of the signal is situated. Note that the apodization factor is included in all OAM power spectra, while in all the amplitude and intensity plots we present the actually measured amplitude E_{obs} without inserting the apodization factor.

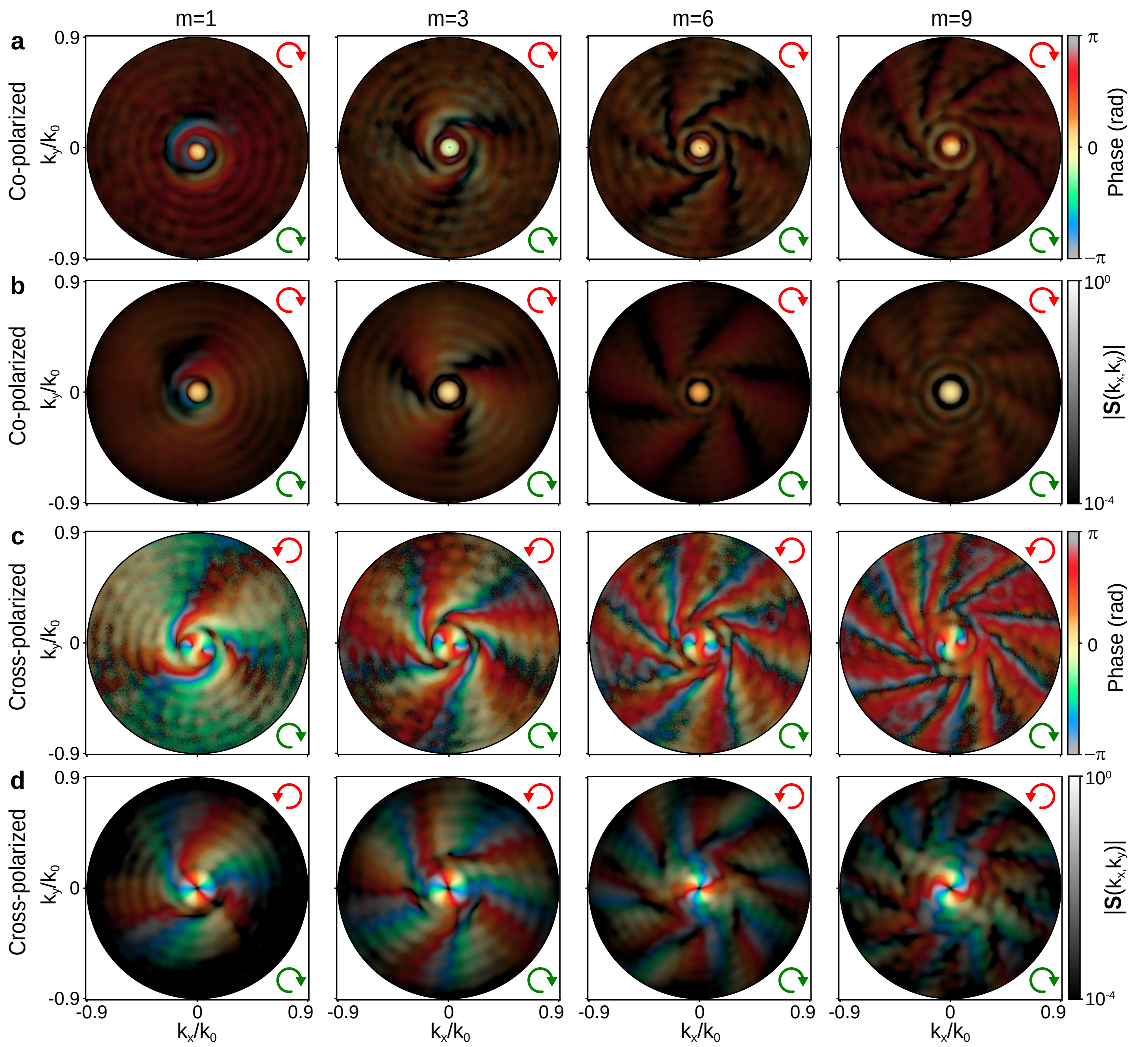

Figure 4. Measured (**a** and **c**) and simulated (**b** and **d**) combined amplitude and phase profiles for $m > 0$ spirals. Field profiles in the Fourier plane of spirals with $m = 1, 3, 6, 9$. The transmitted polarization channels are co- (**b** - **c**) and cross-polarized (**c** - **d**), with RHC polarized input. The plots show a combined representation of phase as hue and field amplitude as brightness (logarithmic scale). Each amplitude profile is normalized by its maximum. The green and red arrows indicate the input and output polarizations, respectively.

III Additional OAM decomposition results

With the full k-resolved field at hand, one can computationally determine the θ-resolved OAM density or in fact project the field on any desired basis function set. This is a large advantage over other reported measurement schemes to determine OAM in paraxial beams, which rely on sequential measurements of integrated transmission through a combined set of spatial filters (to select θ) and (digital) holograms (to select a target $e^{il\varphi}$) [4, 5]. We report such results by plotting the polar-angle-resolved integrand of Eq. 5 in the main text, namely $(C_l(\theta))^2 \cdot \sin(\theta)$ normalized by $\sum_{l=-\infty}^{\infty} p_l$ in Fig. S 5 a) and b) for a $m = -5$ spiral measured in co- and cross-polarization. We find that the main contribution to the OAM density at around $\theta = 0°$ is the OAM mode $l = 0$, which is partially suppressed in cross-polarization in favor of $l = +2$. The high directionality of these two OAM modes can be explained by transmission of the weakly focused excitation light through the gold film. The presence of the OAM mode $l = 0$ in cross-polarization is likely due to the non-perfect extinction ratio of the polarizers. As the aperture itself is sub-wavelength it does radiate at off-center angles giving rise to contributions at $l = 0$ in the co-polarized and $l = +2$ in the cross-polarized channel. For co-polarized scattering, shown in Fig. S 5 a), the maximal OAM density at $l = -5$ is at around $\theta = 18.9°$. For the cross-polarized case shown in Fig. S 5 b) the maximal OAM density $l = -3$ is scattered at an angle $\theta = 16.4°$ and the maximal OAM density value of the parasitic OAM mode $l = 2$ at $\theta = 4.8°$. In general, for the spiral nanostructures studied here, we find that the leakage channels with $l = 0$ and $l = \pm 2$ appear at lower angles than the desired, groove-number (m) dependent OAM modes.

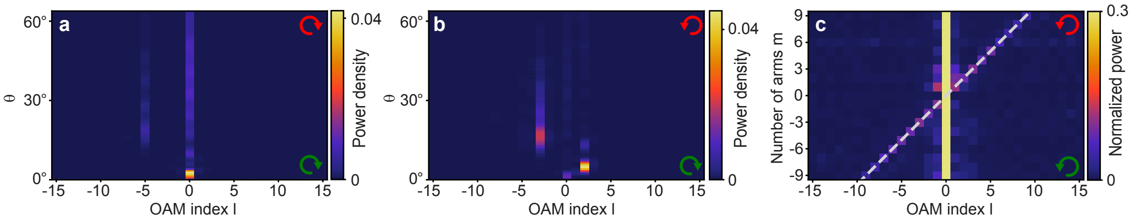

Figure 5. θ-resolved OAM density spectra for a spiral with $m = -5$ in co- (**a**) and cross-polarization (**b**) with RHC polarized input. (**c**) OAM power spectra as a function of m in co-polarization with LHC polarized input. The dashed line indicates $l = m$. The green and red arrows indicate the input and output polarizations, respectively.

IV Description of validation and redundancy removal approaches

Using the well-established polarimetry technique provides us access to wavevector-resolved phase differences between orthogonally polarized fields, as described in the main text. In Eq. 6 in the main text three different combinations of S_1, S_2 and S_3 are shown and expressed in terms of phase differences between vertical and horizontal, anti-diagonal and diagonal and LHC and RHC polarizations. As an example, the main part of the paper examines Eq. 6 a) using polarimetrically and holographically determined phase differences between the vertical and horizontal polarizations for a $m = -5$ spiral, see Fig. 6 a). Here, the two remaining consistency checks are shown in accordance to the Eqs. 6 b) and c), which again show a good visual match, see Fig. S 6. As mentioned in the main text, off-axis holography contains an arbitrary phase offset. Here, in order to make the two plots easily comparable, the phase offset is set automatically by minimizing the integrated difference between the phase difference profiles retrieved from holography and polarimetry [6].

Furthermore, the main text discusses the possibility of utilizing the information redundancy present in combined polarimetric and holographic approaches to reconstruct phase profiles in polarization channels, in which holographic measurements have not been performed. This becomes evident when taking a look

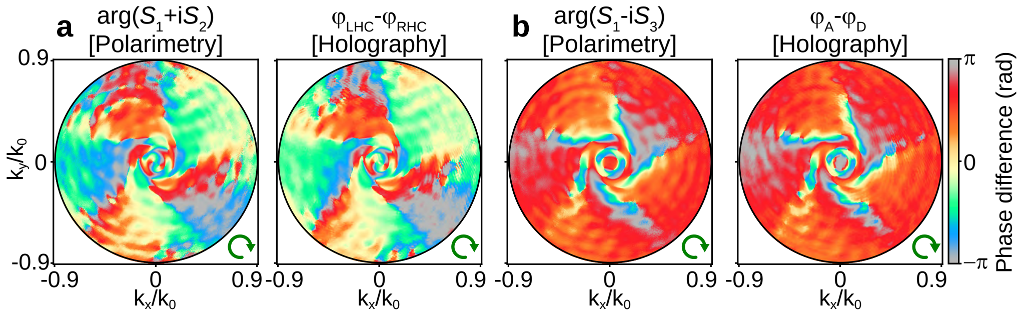

Figure 6. Validation of holography using polarimetric phase difference maps. Difference between phase maps with LHC and RHC (**a**) and anti-diagonal and diagonal (**b**) outgoing polarizations respectively retrieved using polarimetry and holography for RHC polarized input light and a $m = -5$ spiral. The green arrows indicate the input polarization.

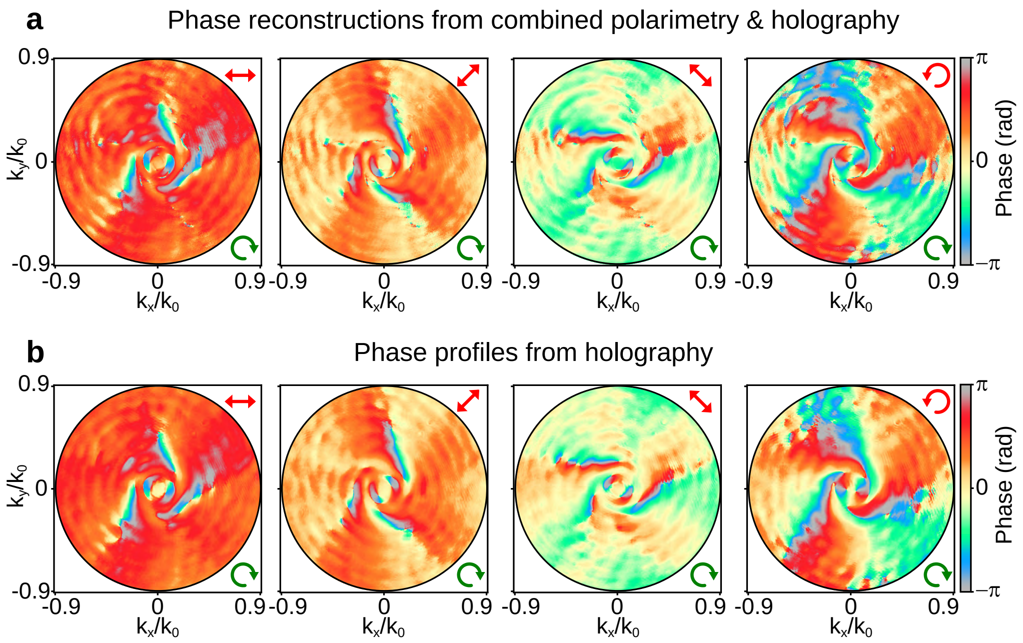

Figure 7. Phase profiles from minimally redundant, combined holography and polarimetry approach. Phase profiles in four polarization channels retrieved using a combination of polarimetry and holography (**a**) with correspondent purely holographic phase profiles for comparison (**b**). The input polarization is RHC and a $m = -5$ spiral is used. The green and red arrows indicate the input and output polarizations, respectively.

at the electric field amplitudes in the horizontal and vertical polarization basis:

$$E_H = E_x \exp(i\varphi_H) \qquad E_V = E_y \exp(i\varphi_V)$$
$$E_D = \frac{1}{\sqrt{2}}\left[E_x \exp(i\varphi_H) + E_y \exp(i\varphi_V)\right] \qquad E_A = \frac{1}{\sqrt{2}}\left[E_x \exp(i\varphi_H) - E_y \exp(i\varphi_V)\right]$$
$$E_{RHC} = \frac{1}{\sqrt{2}}\left[E_x \exp(i\varphi_H) - iE_y \exp(i\varphi_V)\right] \qquad E_{LHC} = \frac{1}{\sqrt{2}}\left[E_x \exp(i\varphi_H) + iE_y x \exp(i\varphi_V)\right], \tag{5}$$

where φ_H and φ_V are the phase profiles in the x-direction and y-direction, respectively. Note, that since the integration time of the detector is much slower than the electric field oscillation time, the measured

intensities are averaged, scalar quantities $I = \left\langle |\mathbf{E}|^2 \right\rangle$. As evident from Eq. 5, only four quantities (E_x, E_y, φ_H and φ_V) need to be known to reconstruct all other polarization channels. This fact is utilized in our reconstruction procedure described below. First, we use $\arg(S_2 + iS_3)$ in combination with the holographically determined phase profile φ_V to reconstruct φ_H, see Eq. 6 a) in the main article. Then, using these two phase profiles and E_x and E_y, which are also known from polarimetry measurements ($\sqrt{I(0°, 0°)}$ and $\sqrt{I(90°, 90°)}$), we determined the remaining complex electric fields E_H,E_D,E_A,E_{RHC} and E_{LHC} as specified in Eq. 5. From these complex fields we determine the respective phase profiles and compare them to the purely holographic phase profiles, as shown in Fig. 6 b) and Fig. S 7. The input polarization is RHC and a $m = -5$ spiral is used. The phase offset for the combined phase reconstructions is set such that the integrated phase difference between the phase profiles under comparison is minimized.

References

1. Goldstein, D. *Polarized Light, Third Edition* (CRC Press, 2010).
2. Lieb, M. A., Zavislan, J. M. & Novotny, L. Single-molecule orientations determined by direct emission pattern imaging. *J. Opt. Soc. Am. B* **21,** 1210–1215 (2004).
3. Kurvits, J. A., Jiang, M. & Zia, R. Comparative analysis of imaging configurations and objectives for Fourier microscopy. *J. Opt. Soc. Am. A* **32,** 2082–2092 (2015).
4. Litvin, I. A., Dudley, A., Roux, F. S. & Forbes, A. Azimuthal decomposition with digital holograms. *Opt. Express* **20,** 10996–11004 (2012).
5. Schulze, C., Dudley, A., Flamm, D., Duparre, M. & Forbes, A. Measurement of the orbital angular momentum density of light by modal decomposition. *New J. Phys.* **15,** 073025 (2013).
6. Nelder, J. A. & Mead, R. A simplex method for function minimization. *The computer journal* **7,** 308–313 (1965).
